# *Lactiplantibacillus plantarum* ST-III and *Lacticaseibacillus rhamnosus* KF7 Enhance the Intestinal Epithelial Barrier in a Dual-Environment In Vitro Co-Culture Model

**DOI:** 10.3390/microorganisms12050873

**Published:** 2024-04-26

**Authors:** Yilin Zhang, Rachel C. Anderson, Chunping You, Ajitpal Purba, Minghui Yan, Paul Maclean, Zhenmin Liu, Dulantha Ulluwishewa

**Affiliations:** 1 State Key Laboratory of Dairy Biotechnology, Shanghai Engineering Research Center of Dairy Biotechnology, Dairy Research Institute, Bright Dairy & Food Co., Ltd., Shanghai 200436, China; yilin027@outlook.com (Y.Z.); youchunping@brightdairy.com (C.Y.); yanminghui@sibcb.ac.cn (M.Y.); 2 AgResearch, Te Ohu Rangahau Kai, Palmerston North 4410, New Zealand; rachel.anderson@agresearch.co.nz (R.C.A.); ajitpal.purba@agresearch.co.nz (A.P.); 3 AgResearch, Grasslands Research Centre, Palmerston North 4410, New Zealand; paul.maclean@agresearch.co.nz

**Keywords:** Caco-2 cells, TEER, tight junctions, probiotics, intestinal barrier integrity

## Abstract

Intestinal barrier hyperpermeability, which is characterised by impaired tight junction proteins, is associated with a variety of gastrointestinal and systemic diseases. Therefore, maintaining intestinal barrier integrity is considered one of the effective strategies to reduce the risk of such disorders. This study aims to investigate the potential benefits of two probiotic strains (*Lactiplantibacillus plantarum* ST-III and *Lacticaseibacillus rhamnosus* KF7) on intestinal barrier function by using a physiologically relevant in vitro model of the intestinal epithelium. Our results demonstrate that both strains increased transepithelial electrical resistance, a measure of intestinal barrier integrity. Immunolocalisation studies indicated that this improvement in barrier function was not due to changes in the co-localisation of the tight junction (TJ) proteins ZO-1 and occludin. However, we observed several modifications in TJ-related genes in response to the probiotics, including the upregulation of transmembrane and cytosolic TJ proteins, as well as TJ signalling proteins. Gene expression modulation was strain- and time-dependent, with a greater number of differentially expressed genes and higher fold-change being observed in the *L. plantarum* ST-III group and at the latter timepoint. Further studies to investigate how the observed gene expression changes can lead to enhanced barrier function will aid in the development of probiotic foods to help improve intestinal barrier function.

## 1. Introduction

The human intestinal epithelium is a single-cell layer that separates the intestinal lumen from the internal milieu [1]. The epithelium acts as a dynamic barrier that prevents the entry of antigens and pathogens into the body, helping maintain immune tolerance, whilst simultaneously enabling the permeability of essential ions, nutrients, and water. A key component of the epithelial barrier are tight junction (TJ) proteins that seal the paracellular spaces between the cells [2]. Thus, proper regulation of the TJ proteins is crucial for maintaining epithelial barrier integrity. When epithelial barrier function is compromised, abnormal antigen exposure caused by increased paracellular permeability can disrupt the balance between immune tolerance and activation. In line with this, a leaky intestinal barrier, characterised by compromised TJs, has been implicated in gastrointestinal disorders (such as inflammatory bowel disease [3], irritable bowel syndrome [4], and celiacs disease [5]), as well as systemic disorders (such as type 1 diabetes and multiple sclerosis) [6]. Therefore, there is growing interest in strategies to help maintain intestinal barrier integrity and in turn reduce the risk of such conditions.

One promising strategy for enhancing intestinal barrier function involves the use of probiotics. Specifically, strains of lactobacilli have been shown to strengthen the epithelial barrier via modulating the epithelial TJs [7]. For example, strains of *Lactiplantibacillus plantarum* and *Lacticaseibacillus rhamnosus* have been shown to improve intestinal barrier integrity and protect against pathogen-mediated disruptions of the barrier in vitro [8,9]. Strains of *L. plantarum* have also been shown to upregulate expression of the TJ-related genes and proteins, such as occludin and zona occludens-1 (ZO-1), both in vivo and in vitro [10,11]. Moreover, *L. plantarum* has been shown to modulate the localisation of these TJ proteins in vitro as well as in the duodenum of healthy human volunteers [12]. In a similar vein, *L. rhamnosus* was able to prevent H_2_O_2_-induced redistribution of TJ proteins in vitro [13].

Our aim was to investigate the potential benefits of two probiotic strains in a physiologically relevant model of the intestinal epithelium. These were *L. plantarum* ST-III, a strain isolated from traditional Chinese pickle [14], and *L. rhamnosus* KF7, a strain isolated from traditional fermented milk called Kefir from the Yunnan province of China [15]. Previous research has shown that *L. plantarum* ST-III can protect against *Salmonella* invasion by increasing expression of the TJ proteins occludin and ZO-1 in the colon in a mouse model [16]. The ability of *L. rhamnosus* KF7 [15] to ameliorate intestinal barrier dysfunction is largely unexplored. In addition, the effects of the two probiotic strains on a healthy intestinal barrier have not been studied.

We hypothesised that these lactobacilli strains can maintain and strengthen intestinal barrier function in an in vitro model of the intestinal epithelium by modulating TJ-related genes and proteins. To accurately test this hypothesis, it was important to ensure that the oxygen in the growth environment of the strains mimicked the physiological environment in vivo. Lactobacilli are considered oxygen-tolerant anaerobes [17]; although they can survive under aerobic conditions, these probiotics express differentially abundant proteins and transcripts when cultured under aerobic versus anaerobic conditions [18,19]. Thus, it is plausible that the effects of such bacteria on intestinal epithelial cells will be different when in the oxygen-deficient environment of the intestinal lumen, compared to when cultured in vitro under conventional aerobic conditions. To overcome this limitation, we employed a previously validated apical–anaerobic model of the intestinal epithelial barrier [20]. The model utilises a human colorectal adenocarcinoma (Caco-2) cell line, as these cells spontaneously differentiate into polarised intestine enterocyte-like cells, characterised by TJs between adjacent cells [21,22,23]. Differentiated Caco-2 monolayers are cultured in a proprietary dual-environment co-culture system, such that the apical side of the cells, representing the intestinal lumen, is anaerobic, analogous to the environment of the colon. Bacteria are co-cultured in the apical–anaerobic side of the monolayer, while the oxygen in the basal side of monolayer ensured the survival of the oxygen-requiring Caco-2 cells. As the probiotics are co-cultured in an oxygen-deficient environment similar to the intestinal lumen, this approach is arguably more physiologically relevant than conventional models where probiotics are co-cultured with epithelial cells in an aerobic atmosphere.

## 2. Methods

### 2.1. Mammalian Cell Culture

Caco-2 cells (HTB37) were obtained from the American Type Culture Collection at passage 18 and used in experiments at passages 28–32. Cell cultures were maintained in Gibco Medium 199 (M199; Thermo Fisher Scientific, Waltham, MA, USA) containing 10% (*v*/*v*) foetal bovine serum (FBS; Moregate BioTech, Hamilton, New Zealand) and 1% (*v*/*v*) MEM non-essential amino acids 100× solution (NEAA; Thermo Fisher Scientific) at 37 °C in a 5% CO_2_ humidified environment. For co-culture experiments, Caco-2 cells were seeded on Transwell polyester filter inserts (6.5 mm, 0.4 µm pore; Corning Inc., Corning, NY, USA) at a density of 8 × 10^4^ cells per insert and cultured for 17 days prior to the co-culture experiment.

### 2.2. Bacterial Strains and Growth Conditions

*L. plantarum* ST-III (CGMCC No. 0847) and *L. rhamnosus* KF7 (CGMCC No. 6430) were supplied by Bright Dairy & Food Co., Ltd. (Shanghai, China) and were stored at −80 °C in De Man-Rogosa-Sharp (MRS) broth containing 50% (*v*/*v*) glycerol. Bacteria were grown on MRS agar (Oxoid, Basingstoke, UK) at 37 °C in an anaerobic workstation (Whitley A85 Workstation, Don Whitley Scientific, Bingley, UK) with an atmosphere of 10% CO_2_, 10% H_2_ in N_2_. Bacteria on agar plates were stored up to 3 weeks at 4 °C. Liquid cultures of bacteria were prepared by inoculating 5 mL of anaerobic MRS broth (Merck, Darmstadt, Germany) with a single colony from an agar plate and incubating overnight at 37 °C in an anaerobic workstation. Anaerobic culture medium was prepared by placing an open vial of culture medium inside an anaerobic workstation (atmosphere of 10% CO_2_, 10% H_2_ in N_2_; Whitley A85 Workstation, Don Whitley Scientific) overnight.

### 2.3. Dual-Environment Co-Culture System

The dual-environment co-culture system used for co-culture experiments has been described elsewhere [24]. Briefly, the system utilised a proprietary co-culture chamber inside a modified anaerobic workstation. Each well of the co-culture chamber contained 3 mL of (aerobic) cell culture medium (M199 containing 1% (*v*/*v*) NEAA and 10% (*v*/*v*) FBS) and was fitted with a Transwell insert containing a differentiated Caco-2 monolayer and 260 µL of anaerobic cell culture medium (M199 containing 1% (*v*/*v*) NEAA). FBS was omitted from the apical culture medium to prevent potential overgrowth of bacteria during co-culture experiments. The Transwell insert separated the aerobic environment of the well from the anaerobic environment of the anaerobic workstation. This enabled the culture of Caco-2 cells such that they were exposed to an anaerobic environment on the apical side, while the diffusion of dissolved oxygen from the chamber well via the semi-permeable membrane ensured their survival [20].

### 2.4. Co-Culture of Mammalian and Bacterial Cells under Apical–Anaerobic Conditions

The day prior to co-culture experiments, the medium in Transwell inserts containing differentiated Caco-2 monolayers was replaced with 260 µL of M199 with 1% (*v*/*v*) NEAA. On the day of the co-culture experiment, 3 mL aerobic cell culture medium was added to each well of the co-culture chamber, and each well was subsequently fitted with a Transwell insert containing a differentiated Caco-2 monolayer, before transferring the co-culture chamber into the anaerobic workstation. Caco-2 monolayers were incubated under apical–anaerobic conditions for approximately one hour prior to co-culture with probiotics.

Ten µL of overnight bacterial cultures were diluted in 5 mL of anaerobic MRS broth and cultured for 15 h inside an anaerobic workstation until the stationary phase was reached. Following centrifugation at 2492× *g* for 20 min (A-4-81 rotor, Eppendorf 5810 R centrifuge), cultures were resuspended in anaerobic cell culture medium inside an anaerobic workstation. The number of bacteria in the solution was estimated by counting the bacterial cells in a Petroff-Hausser counting chamber (Hausser Scientific, Horsham, PA, USA), following which the medium in the Transwell insert in the dual-environment co-culture system was replaced with bacteria at a multiplicity of infection (MOI) of 1000 in a total volume of 260 µL of anaerobic cell culture medium. To achieve this MOI, probiotics were prepared at a concentration of 1.4 × 10^8^ bacteria/mL. For untreated control monolayers, the medium in the Transwell insert was replaced with 260 µL of anaerobic cell culture medium without bacteria.

### 2.5. Transepithelial Electrical Resistance Assay

Transepithelial electrical resistance (TEER) across Caco-2 monolayers was measured using electrodes built-in to the co-culture chamber connected to a cellZscope controller (nanoAnalytics, Münster, Germany) and software (v 4.3.1; nanoAnalytics). The baseline TEER was measured immediately prior to the addition of treatment. The change in TEER across each monolayer was calculated using the following formula: change in TEER (%) = TEER (Ω.cm^2^)/baseline TEER (Ω.cm^2^) × 100 − 100 (%). Only Caco-2 monolayers where both the baseline TEER and TEER 30 min post-treatment/challenge was >500 Ω.cm^2^ were considered for statistical analysis, as an initial TEER value ≤ 500 Ω.cm^2^ suggests that barrier integrity of the Caco-2 monolayer was compromised prior to the addition of treatment. Three assays (with a total of 14–17 replicates/treatment) were carried out where the TEER was measured every hour for 12 h with or without probiotic treatment.

### 2.6. Analysis of Tight Junction Protein Localisation

For visualization of TJs, Caco-2 monolayers were co-cultured with or without probiotic treatment for eight hours. Following treatment, monolayers were washed with PBS, fixed in 4% (*w*/*v*) paraformaldehyde for 15 min, and permeabilised in PBS containing 0.2% (*v*/*v*) Triton X-100, 1% (*v*/*v*) normal goat serum, and 0.1% (*w*/*v*) sodium azide. The monolayers were incubated overnight at 4 °C in 1 μg/mL of polyclonal rabbit anti-occludin (Thermo Fisher Scientific) and monoclonal mouse anti-ZO-1 (Thermo Fisher Scientific), washed five times in PBS containing 0.1% (*v*/*v*) Triton X-100 to remove non-specific binding, and incubated for 2 h at room temperature in 8 μg/mL of Alexa Flour 555 conjugated goat anti-rabbit IgG (Thermo Fisher Scientific) and Alexa Flour 488 conjugated goat anti-mouse IgG (Thermo Fisher Scientific). The monolayers were washed five times in PBS containing 0.1% (*v*/*v*) Triton X-100 and two times in PBS, following which the Transwell membranes with the Caco-2 monolayers were excised from the plastic supports and mounted on to slides using Prolong gold antifade reagent with DAPI (Thermo Fisher Scientific). Stacks of confocal images were obtained using a Zeiss LSM900 with an Airyscan 2 super-resolution microscope with a 60× oil objective (Manawatu Microscopy and Imaging Centre, Palmerston North, New Zealand).

Co-localisation analysis was performed using the Coloc2 plugin in Fiji (ImageJ version 1.54d) [25]. For each stack (*n* = 6 confocal image stacks per treatment; 15–26 images per stack) the Alexa Flour 555 and Alexa Flour 488 channels were separated, and background subtraction was completed using a rolling ball radius set at 50.0 pixels. Threshold regression was set to Costes (10 Costes randomisations selected) and the point spread function (PSF) was set to 3.0.

### 2.7. Semi-Quantification of Tight Junction Proteins

Semi-quantitative analysis of tight junction proteins was performed on 2–3 different regions of 2 sections per treatment (*n* = 6 confocal images/treatment/protein). The mean fluorescence intensity (mean grey value), representing the level of specific protein expression, was measured by Fiji software (ImageJ version 1.54f) (threshold was set to default).

### 2.8. Gene Expression Analysis

To analyse the effect of probiotics on Caco-2 cell gene expression, co-culture experiments were performed twice, and for each treatment group RNA was extracted from three samples at two timepoints (total *n* = 6 replicates/treatment/timepoint). At each timepoint, following stabilisation of RNA using RNAprotect Cell Reagent (Qiagen, Hilden, Germany), total RNA was extracted using an RNeasy mini kit (Qiagen) as per the manufacturer’s protocol. The RNA quantity and purity were determined using a Nanodrop ND-1000 spectrophotometer (Nanodrop Technologies, Wilmington, DE, USA). Approximately 1000 ng of RNA was used to determine the absolute counts of 90 human TJ-related genes and 6 reference genes (nCounter Plexset Preselected Human Tight Junction Pathway Panel) using NanoString nCounter technology [26]. This method utilises molecular barcodes on gene-sequence-specific probes and single-molecule imaging to count RNA copies. Briefly, RNA samples were hybridised by adding 13.5 μL of MasterMix and 1.5 μL of RNA per tube of a 12-tube strip immediately before placing the strip at 67 °C for 22 h. After hybridization, samples were transferred to the nCounter Prep Station to be purified and immobilised in the glass cartridge. For data acquisition, the glass cartridge was then transferred to the nCounter Digital Analyzer which counted and tabulated colour codes on the surface of the cartridge for each target molecule. Data were retrieved from the Analyzer as raw data (Reporter Code Count, RCC) files.

For analysis, RCC files were imported into nSolver Analysis Software (version 4.0.70; NanoString Technologies Inc., Seattle, WA, USA) and underwent the software’s sample quality control routine set to the following criteria: (1) imaging: fields of view registration < 75%; (2) binding density outside the 0.05 to 2.25 range; (3) positive control linearity: positive control R^2^ value < 0.95; and (4) positive control limit of detection: 0.5 fM positive control ≤ 2 standard deviations above the mean of the negative controls. All samples used for statistical analysis passed the quality control routine. After quality assurance, the data were sequentially normalised to the geometric mean of the respective internal positive controls and reference genes (*ABCF1*, *GUSB*, *HPRT1*, *LDHA*, *POLR1B*, *RPLP0*).

### 2.9. Statistical Analysis

For the TEER assay data, the effect of treatment on change in TEER over time was compared using a mixed-model analysis of variance to account for the fact that the same monolayers were measured over time. Models were fitted by the restricted maximum-likelihood (REML) method using the nlme package (version 3.1-164) [27] in R (version 4.3.2) [28]. The statistical model included the effect of treatment, time, and their interaction as fixed effects, and the Transwell inserts nested within blocks (where one run of an experiment was considered a block) as a random effect. If the treatment × time interaction was significant (*p* < 0.05), pairwise comparisons were applied using estimated marginal means using the emmeans package (version 1.10.0) [29]. The false discovery rate (q) was applied to the tests of the marginal means, with differences considered significant when q < 0.05.

For the tight junction protein co-localisation data, the Pearson’s correlation value between the two channels was transformed using the Fishers Transformation to normalise the data, and treatments were compared by performing the analysis of variance test using the aov () function in R. Differences were considered statistically significant when the *p* value < 0.05.

For tight junction protein quantification, differences between groups were compared by independent samples Student’s *t*-test (SPSS22.0 software, SPSS Inc., Chicago, IL, USA). A non-parametric test (Mann–Whitney U test) was used to compare the difference in th emean fluorescence intensity of occludin proteins between the ‘no treatment’ group and ‘ST-III’ group since the data did not conform to a normal distribution. Differences were considered statistically significant when the *p* value < 0.05.

For the gene expression data, statistical analysis was performed using R [28] version 4.3.0. Partial least squares-discriminant analysis (PLS-DA) was performed using the mixOmics R package version 6.24.0 [30]. For each individual gene, permutation ANOVAs were performed on the normalised counts using the lmPerm package version 2.1.0 [31], and post hoc tests were performed using the predictmeans package (version 1.0.9) [32]. Within each timepoint, each probiotic treatment was compared to the no-treatment control group to generate a fold change to correspond to the post hoc *p*-values. These data were then mapped to the Kyoto Encyclopedia of Genes and Genomes (KEGG) pathways database [33] using the KEGG mapping tools [34].

## 3. Results

### 3.1. Epithelial Barrier Function Was Maintained in the Apical–Anaerobic Model

**Figure 1:**
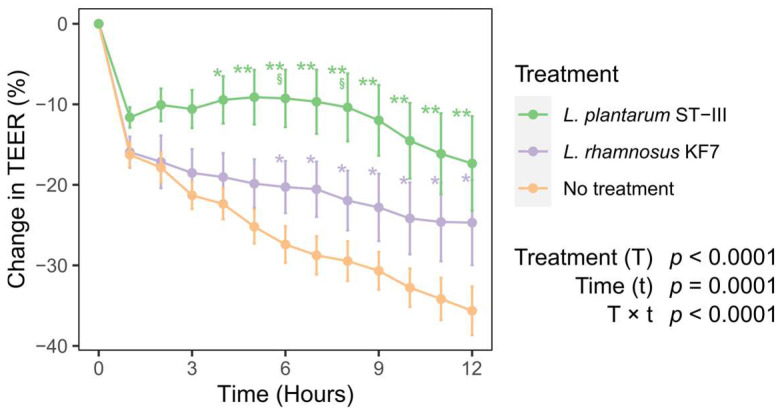
Effect of probiotic treatment on TEER across Caco-2 monolayers over time. Seventeen-day-old differentiated Caco-2 monolayers were co-cultured with *Lactiplantibacillus plantarum* ST-III or *Lacticaseibacillus rhamnosus* KF7 with a multiplicity of infection of 1000 or cultured without probiotic bacteria (no treatment; control). Graph shows mean (+/− SEM) change in TEER (n = 14–17 per treatment) * q < 0.05, ** q < 0.01, compared to control; § q < 0.05 compared to *L. rhamnosus* KF7 treatment.

We investigated the effects of *L. plantarum* ST-III and *L. rhamnosus* KF7 on epithelial barrier function in vitro by measuring TEER as an indicator of intestinal epithelial cell TJ integrity in an apical–anaerobic model of the intestinal epithelium. The TEER dropped by between 11.6 ± 4.4 and 16.2 ± 3.7% (estimated marginal mean (emmean) ± standard error (SE)), relative to baseline (0 h), within the first hour, in all treatment groups, including the no-treatment control group (Figure 1). This initial drop in TEER is frequently observed when Caco-2 monolayers are transferred into the dual-environment co-culture system and is likely an acclimatation of the cells to the apical–anaerobic conditions. In the control monolayers, the TEER continued to decrease throughout the assay, leading to a change in TEER of 19.4 ± 2.2% (emmean ± SE) between 1 and 12 h (q < 0.001). However, all monolayers in the control group retained a TEER of ≥500 Ω.cm^2^ for the duration of the assay, suggesting that the Caco-2 cells maintain an intact and well-formed barrier for at least 12 h in the apical–anaerobic environment.

### 3.2. Probiotics Improved Transepithelial Electrical Resistance

Treatment with *L. rhamnosus* KF7 mitigated the reduction in TEER observed in control monolayers. Following the initial drop, TEER remained stable between 1 and 6 h. However, there was a statistically significant change in TEER between the 1 h and 7 h timepoints (4.6 ± 2.2% (emmean ± SE); q < 0.05). Nevertheless, Caco-2 monolayers treated with *L. rhamnosus* KF7 had a higher TEER than that of control Caco-2 monolayers between 6 and 12 h (Figure 1).

Monolayers treated with *L. plantarum* ST-III did not show any change in TEER following the initial drop at 1 h, indicating *L. plantarum* ST-III is able to eliminate the drop in TEER observed in untreated monolayers. *L. plantarum* ST-III treated monolayers had a higher TEER compared to control monolayers between 4 and 12 h (Figure 1). Moreover, *L. plantarum* ST-III-treated Caco-2 monolayers had a higher TEER than *L. rhamnosus* KF7-treated Caco-2 monolayers at 6 h and 8 h. Overall, the data indicate that treatment with the probiotics improved the barrier integrity of Caco-2 monolayers cultured in apical–anaerobic conditions.

### 3.3. Co-Localisation of Occludin and Zona Occludens-1 Was Not Affected by Probiotic Treatment

**Figure 2:**
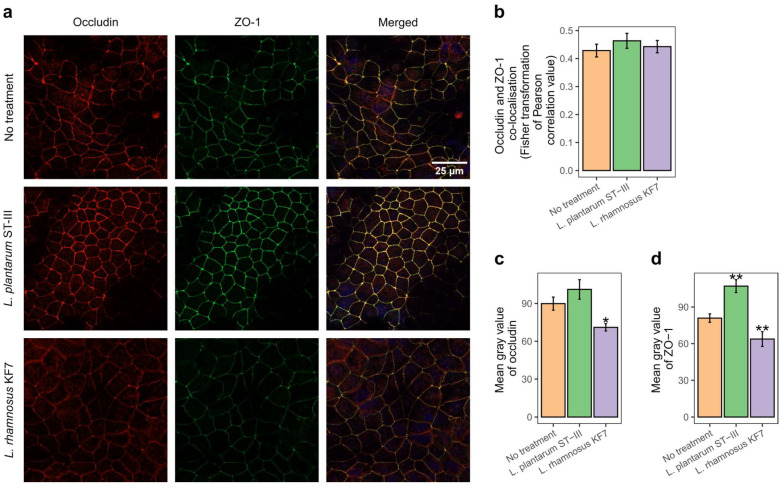
Tight junction protein abundance and localisation. (**a**) Immunolocalisation of tight junction proteins occludin (red) and zona occludens-1 (ZO-1; green) in 17-day-old Caco-2 monolayers co-cultured with the indicated probiotic strains (multiplicity of infection of 1000) for 8 h. Scalebar, 25 µm. (**b**) Effect of probiotics on co-localisation of occluding and ZO-1 in Caco-2 cell monolayers. Graph shows the mean (+/− SEM) correlation (*n* = 6 confocal image stacks per treatment; 15–26 images per stack). (**c**) Effect of probiotics on abundance of occludin. Graph shows the mean (+/− SEM) grey value of occludin (*n* = 4 confocal image stacks per treatment). (**d**) Effect of probiotics on abundance of ZO-1. Graph shows the mean (+/− SEM) grey value of ZO-1 (*n* = 4 confocal image stacks per treatment). * *p* < 0.05, ** *p* < 0.01 compared to ‘no treatment’.

The TJ proteins occludin and zona occludens-1 (ZO-1) were visualised using confocal microscopy to help determine whether the observed effects on TEER were due to changes in the localisation of these proteins. Typical images for each treatment group are shown in in Figure 2a. Both proteins localised at the cell boundaries regardless of the treatment group, with very little internalised protein. The mean Pearson’s correlation values for co-localisation of occludin and ZO-1 were weak (below 0.5) across all treatment groups (Figure 2b). The treatment effect was not significant (*p* = 0.595) indicating that the co-localisation of the two TJ proteins was not affected by *L. plantarum* ST-III or *L. rhamnosus* KF7.

### 3.4. Probiotics Modulated Occludin and Zona Occludens-1 Protein Abundance

The mean fluorescence intensity (grey value) of the confocal images were analysed to estimate the abundance of occludin (Figure 2c) and ZO-1 (Figure 2d) protein in each treatment group. *L. plantarum* ST-III appeared to have no effect on occludin abundance but increased ZO-1 abundance (*p* < 0.01). Conversely, treatment with *L. rhamnosus* KF7 caused a reduction abundance of both occludin (*p* < 0.05) and ZO-1 (*p* < 0.01).

### 3.5. Effect of Probiotics on Tight Junction Gene Expression Was Both Strain- and Time-Dependent

To help identify the mechanisms by which *L. rhamnosus* KF7 and *L. plantarum* ST-III improved TJ integrity, we analysed the expression of 90 genes involved in the TJ signalling using NanoString technology (Supplementary Table S1). Gene expression was analysed at both 4 h and 8 h following treatment with probiotics. A timepoint of 4 h was chosen because it was at this timepoint that probiotic-treated Caco-2 monolayers started to show improvements in barrier function compared to untreated monolayers. An 8 h timepoint was chosen as distinct differences in TEER could be observed between all three treatment groups at this timepoint.

**Figure 3:**
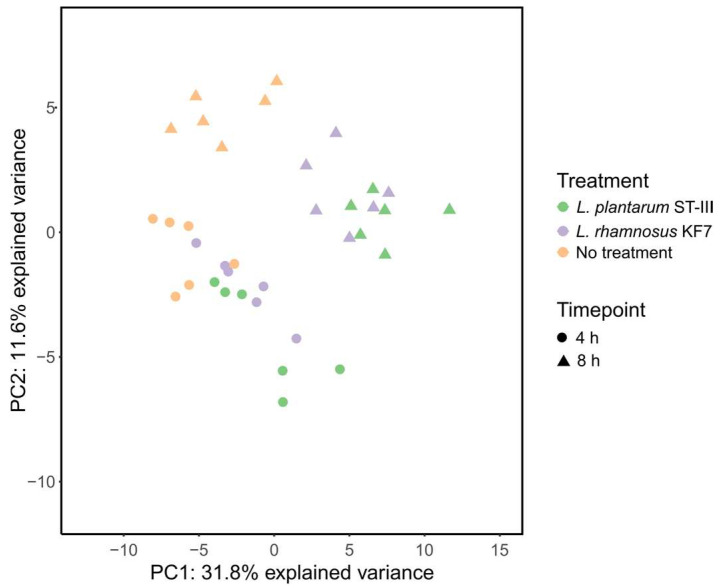
Partial least squares-discriminant analysis (PLS-DA) of gene expression profiles of untreated Caco-2 monolayers (no treatment) and Caco-2 monolayers co-cultured with *L. plantarum* ST-III or *L. rhamnosus* KF7 at 4 h and 8 h.

PLS-DA could discriminate between the 4 h and 8 h samples (R^2^: 0.897, Q^2^: 0.809, Figure 3). For each timepoint, probiotic-treated samples tended to cluster closer together, separate from the control samples. This indicates that the gene expression profiles of the monolayers treated with *L. rhamnosus* KF7 and *L. plantarum* ST-III were more similar to each other than that of the untreated monolayers at the respective timepoint. The separation between the probiotic-treated and control monolayers was larger in the 8 h samples, indicating that the probiotics had a bigger effect on Caco-2 gene expression following 8 h of co-culture compared to 4 h of co-culture.

**Figure 4:**
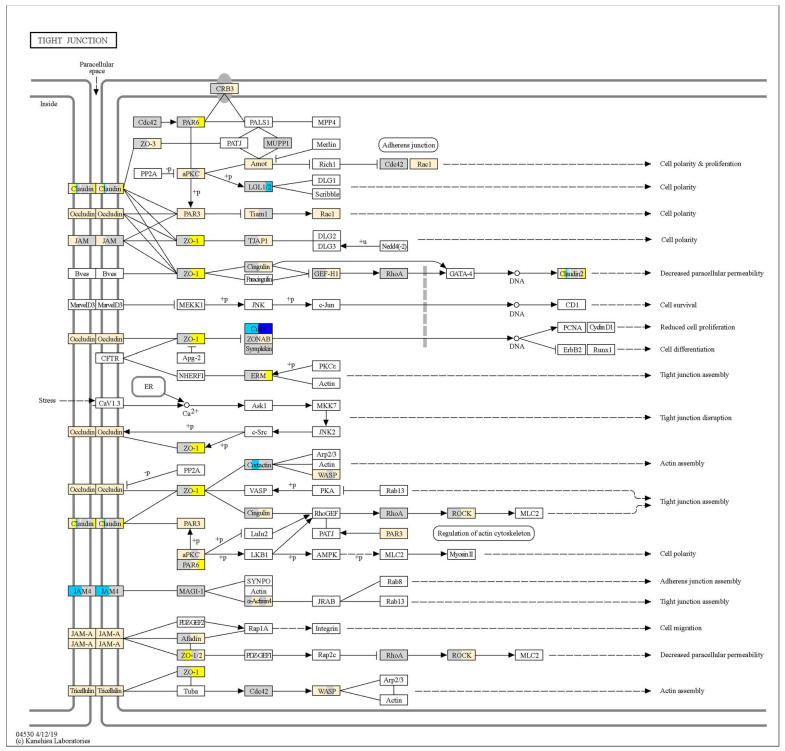
Tight junction signalling in response to *L. plantarum* ST-III. Transcriptional information has been projected onto the KEGG tight junction pathway. For each gene, the left half represents the 4 h timepoint, and the right half represents the 8 h timepoint. Genes with increased expression in Caco-2 cells co-cultured with bacteria (compared to untreated Caco-2 cells at the given timepoint) are depicted in yellow (*p* < 0.05), decreased expression depicted in blue (*p* < 0.05), and no change in expression (*p* > 0.05) depicted in grey. Fold-changes > 1.5 or <−1.5 are more intensely coloured. Pathway is a copyright of Kanehisa Laboratories, Kyoto University, Japan.

**Figure 5:**
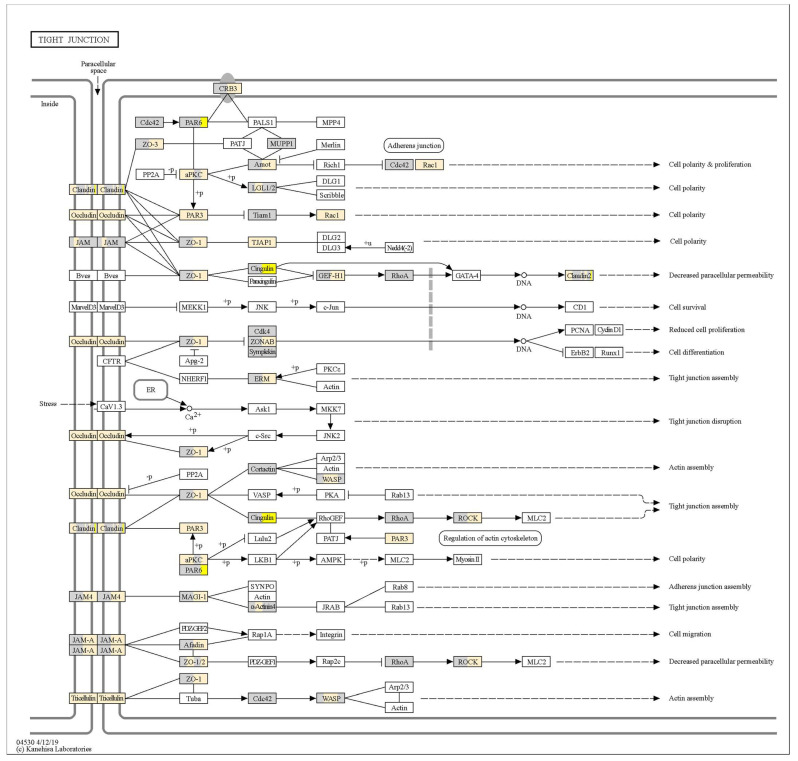
Tight junction signalling in response to *L. rhamnosus* KF7. Transcriptional information has been projected onto the KEGG tight junction pathway. For each gene, the left half represents the 4 h timepoint, and the right half represents the 8 h timepoint. Genes with increased expression in Caco-2 cells co-cultured with bacteria (compared to untreated Caco-2 cells at the given timepoint) are depicted in yellow (*p* < 0.05), decreased expression depicted in blue (*p* < 0.05), and no change in expression (*p* > 0.05) depicted in grey. Fold-changes >1.5 or <−1.5 are more intensely coloured. Pathway is a copyright of Kanehisa Laboratories, Kyoto University, Japan.

**Figure 6:**
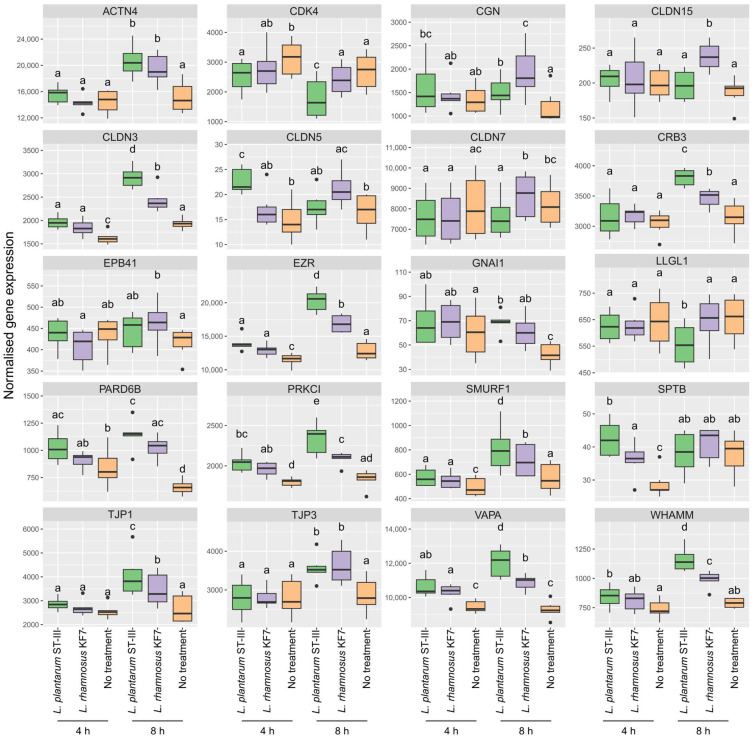
Gene expression in untreated Caco-2 cells and Caco-2 cells co-cultured with *L. plantarum* ST-III or *L. rhamnosus* KF7 at 4 h and 8 h. Box and whisker plots indicate the median (middle line), first and third quartile (boundaries of box), and 1.5 times the interquartile range (whiskers). Treatments which do not share the same letter (a, b, c, d, e) are significantly different (*p* < 0.05).

To visualise the effects of probiotics on TJ signalling, gene expression fold-change (relative to no-treatment control monolayers at the respective timepoint) data were projected onto a KEGG ‘Tight junction—*Homo sapiens*’ pathway map. TJ signalling in response to *L. plantarum* ST-III and *L. rhamnosus* KF7 are depicted in Figure 4 and Figure 5, respectively. As shown in these figures, certain genes, such as those encoding occludin and tricellulin, were upregulated by both probiotics. However, several genes, including those encoding ZO-1, cingulin, and ROCK, were upregulated at 8 h but not 4 h following probiotic treatment. Differences could also be observed between the treatments, where, for example, the gene encoding for Cdk4 was downregulated by *L. plantarum* ST-III but not the *L. rhamnosus* KF7 treatment. In fact, of the total 90 genes analysed, 54 genes had a significant ‘treatment’ effect (*p* < 0.05), while 53 genes had a significant ‘time’ effect (*p* < 0.05) (Supplementary Table S1). Twenty genes showed a significant treatment × time interaction (*p* < 0.05) (Figure 6). For example, certain genes, including CRB3 and PRKCI, were expressed at a significantly higher level in the *L. plantarum* ST-III group at the 8 h timepoint compared to all the other treatment groups at either timepoint. Overall, the gene expression data suggest that the two probiotics modulate TJ signalling in a strain- and time-dependent manner.

## 4. Discussion

Consistent with our hypothesis, *L. plantarum* ST-III and *L. rhamnosus* KF7 modulated tight-junction-related genes and proteins and enhanced intestinal barrier function in a physiologically relevant apical–anaerobic in vitro model of the intestinal epithelium. While the epithelial model maintained its barrier integrity for the duration of the TEER assay, we observed a small gradual drop in the untreated control monolayers over the 12-h period. This gradual loss of TEER may be a result of the reduced amount of oxygen available to the Caco-2 cells, as the deprivation of oxygen has been shown to lead to decreased TEER in Caco-2 cells [35]. As this drop in TEER was mitigated by *L. rhamnosus* KF7 but eliminated by *L. plantarum* ST-III, it suggests that the latter is more effective at improving epithelial barrier function. This is also apparent in the fact that *L. plantarum* treatment caused TEER to improve earlier than *L. rhamnosus* did, and that the TEER of the *L. plantarum* treated cells was higher than that of the *L. rhamnosus* treated cells. Consistent with this, *L. plantarum* ST-III, but not *L. rhamnosus* KF7, increased the abundance of ZO-1, a cytoplasmic protein which anchors transmembrane TJ proteins to the actin cytoskeleton, and hence contribute to barrier function [36]. The increased ZO-1 abundance by *L. plantarum* treatment was mirrored in our gene expression data, where although at the 8 h timepoint both probiotic treatments resulted in increased ZO-1 gene expression, a higher fold-change could be observed in the *L. plantarum* treatment.

While we observed changes in TJ protein abundance in response to probiotic treatment, we did not detect any differences in co-localisation of TJ proteins. Previous studies have shown that co-localisation of occludin and ZO-1 decreases when the barrier is leaky due to the internalisation of TJ proteins [37]. Hence our result suggests that the probiotic-induced barrier enhancement was not due to changes in the co-localisation of occludin and ZO-1. Interestingly, both occludin and ZO-1 protein abundance was decreased in *L. rhamnosus* KF7 cells. This was contrary to our expectations, as knock-down of ZO-1 has been shown to reduce TEER [38], whereas we observed an increase in TEER in *L. rhamnosus* KF7 cells. The role of occludin in TEER is less clear, with the overexpression of both wildtype and mutant occludin shown to increase TEER, while mutant occludin also increases paracellular flux, consistent with decreased barrier function [39]. Hence, the improvement in TEER by *L. rhamnosus* KF7 cells is likely due to other TJ signalling modifications as observed in our gene expression analysis results.

The gene expression data were in agreement with the phenotypic TEER data. As shown in the PLS-DA there was greater separation between the untreated and *L. plantarum* ST-III treated samples compared to untreated and *L. rhamnosus* KF7 treated samples at the 4 h timepoint. Thus, the differences in gene expression driving this separation are likely what is causing the increase in TEER caused by *L. plantarum* ST-III (but not *L. rhamnosus* KF7) at the 4 h timepoint. Similarly, greater separation between treatments could be seen in the 8 h samples, which is reflected in the greater TEER differences between treatment groups at the 8 h timepoint.

Treatment with probiotics led to the increase in expression of several genes. Genes encoding the transmembrane proteins occludin and tricellulin were upregulated by both probiotics. Transmembrane proteins mediate cell–cell adhesion and seal the paracellular space between epithelial cells. Occludin and tricellulin are both tetra-span proteins, containing four transmembrane domains [40,41]. However, while occludin seals the space between two adjacent cells, the barrier at the intersection between three epithelial cells is reinforced by tricellulin. Demonstrating the importance of these genes in barrier function, cells expressing truncated occludin mutants have been shown to have increased paracellular permeability, while suppression on the tricellulin gene was shown to impair epithelial barrier integrity [39,41]. Claudins are also tetra-span transmembrane proteins which play a crucial role in the paracellular ionic selectivity of TJs [42]. Several claudins were upregulated by both probiotic strains, and hence, upregulation of claudin may be a mechanism by which the probiotics enhance barrier function. It should also be noted that the increase in occludin and ZO-1 gene expression was inconsistent with the observed decrease in occludin and ZO-1 protein abundance in the *L. rhamnosus* KF7-treated cells. This suggests that the transcripts may not be getting translated or that the protein degradation is occurring faster than the translation of new proteins.

In addition to TJ proteins, expression of genes that encode proteins involved in Rho-signalling (e.g., GEF-H1 [43] and ROCK [44]) were upregulated, indicating that the probiotics may regulate TJ integrity via these pathways. Both strains also upregulated genes encoding for Rac1, PAR3, and aPKC. The latter two genes are involved in the Par3/Par6/aPKC complex, a key molecular complex responsible for cell polarity [45]. Apicobasal polarity is a crucial characteristic of the epithelium and is coupled to TJ formation. Moreover, depletion of PAR3 disrupts TJ assembly, and PAR3 is thought to regulate TJ assembly via interactions with Rac1 [46]. Several related genes were expressed at a significantly higher level in the *L. plantarum* ST-III group at the 8 h timepoint compared to all other treatment groups at either timepoint. These include the genes CRB3 and PRKCI. The protein encoded by CRB3 has been shown to regulate TJs via interactions with Par6 [47], while PrkCi (formerly known as aPKCλ) interacts with Par6 and is involved in the maturation of TJs [48]. The gene encoding for Smurf1, a GTPase regulator that is known to modify epithelial barrier function [49], was also upregulated at a higher level in the *L. plantarum* ST-III group. Hence, it may be via the regulation of these genes that *L. plantarum* ST-III improves barrier function more effectively than *L. rhamnosus* KF7.

Expression of genes encoding for junctional adhesion molecule (JAM) proteins were increased in the *L. plantarum* ST-III treatment group at both the 4 h and 8 h timepoints, while they were only increased at the 8 h timepoint in the *L. rhamnosus* KF7 treatment group. Unlike occludin and tricellulin, JAMs are single-span transmembrane proteins. The appearance of JAMs at cell junctions have been shown to increase TEER as well as decrease paracellular permeability [50]. Moreover, it has recently been deduced that JAM-A and claudins co-ordinately regulate TJ formation as well as cell polarity [51]. Hence the upregulation of JAMs by *L. plantarum* ST-III at the 4 h timepoint may partly be responsible for TEER increase observed in the *L. plantarum* ST-III but not the *L. rhamnosus* KF7 treatment group at the 4 h timepoint.

Overall, the data presented in this study indicate that both *L. plantarum* ST-III and *L. rhamnosus* KF7 enhance epithelial barrier function by modulating TJ signalling. Both strains upregulated genes that encode transmembrane and cytoplasmic TJ proteins. However, as demonstrated in our study, increased gene expression does not mean increased protein abundance, and hence, further studies are necessary to determine the effects of the probiotics on tight junction protein expression. Gene expression modulation was strain- and time-dependent, with a greater number of differentially expressed genes and higher fold-change being observed at the latter timepoint. The gene expression data also provide insights on the possible mechanisms by which each probiotic modulates epithelial tight junction integrity. For example, *L. plantarum* ST-III may provide greater benefits than *L. rhamnosus* KF7 via inducing increased expression of JAM and ZO-1 as well as genes that interact with the Par3/Par6/aPKC complex. Further studies to better understand how the observed gene expression changes can lead to enhanced barrier function will aid in the development of probiotic foods to help improve intestinal barrier function.

## Data Availability

The data supporting the conclusions of this article can be made available by the authors, upon reasonable request.

## Supplementary Materials

The following supporting information can be downloaded at: https://www.mdpi.com/article/10.3390/microorganisms12050873/s1, Supplementary Table S1: Normalised gene expression in Caco-2 cells following 4 h and 8 h of co-culture with or without probiotic treatment.
