# Supplementary material for: Lactiplantibacillus plantarum ST-III and Lacticaseibacillus rhamnosus KF7 Enhance the Intestinal Epithelial Barrier in a Dual-Environment In Vitro Co-Culture Model
Source: Microorganisms. 2024 Apr 26;12(5):873. doi: 10.3390/microorganisms12050873 (PMC11124027; doi:10.3390/microorganisms12050873)
Supplement: Supplementary file 1 [file microorganisms-12-00873-s001.zip › microorganisms-2952295-supplementary.pdf]

Supplementary Table S1. Normalised gene expression in Caco-2 cells following 4 hours and 8 hours of co-culture with or without probiotic treatment.

Gene symbol	Gene name	4 hours[1]			8 hours[1]			P-values[2]		
		L. plantarum ST-III	*L. rhamnosus* KF7	No treatment	*L. plantarum* ST-III	*L. rhamnosus* KF7	No treatment	Treatment	Time	Treatment × Time
**ACTN1**	actinin alpha 1	4660 ± 141 bc	4493 ± 136 abc	4429 ± 134 abc	4799 ± 146 c	4389 ± 133 ab	4152 ± 126 a	0.010	0.425	0.294
**ACTN2**	actinin alpha 2	20 ± 2 ab	23 ± 3 ab	19 ± 2 b	26 ± 3 ab	26 ± 3 a	27 ± 3 a	0.681	0.006	0.608
**ACTN3**	actinin alpha 3	15 ± 2 a	17 ± 2 a	16 ± 2 a	16 ± 2 a	16 ± 2 a	15 ± 2 a	0.823	0.844	0.828
**ACTN4**	actinin alpha 4	15511 ± 694 a	14281 ± 639 a	14361 ± 643 a	20521 ± 918 b	19339 ± 865 b	15012 ± 672 a	0.001	0.000	0.011
**AFDN**	afadin, adherens junction formation factor	2815 ± 159 abc	2632 ± 149 ab	2519 ± 142 a	3012 ± 170 bc	3219 ± 182 c	2546 ± 144 a	0.023	0.047	0.224
**AMOTL1**	angiomotin like 1	5755 ± 216 b	5286 ± 198 ab	4763 ± 179 a	5448 ± 204 b	5231 ± 196 ab	4212 ± 158 c	0.000	0.045	0.316
**ARHGEF2**	Rho/Rac guanine nucleotide exchange factor 2	1337 ± 57 ab	1345 ± 57 ab	1229 ± 52 a	1833 ± 78 c	1832 ± 78 c	1449 ± 62 b	0.001	0.000	0.141
**ASH1L**	ASH1 like histone lysine methyltransferase	1158 ± 85 a	1120 ± 82 a	1132 ± 83 a	1054 ± 77 a	1208 ± 88 a	1030 ± 75 a	0.567	0.517	0.403
**CASK**	calcium/calmodulin dependent serine protein kinase	5398 ± 130 a	5565 ± 134 a	5320 ± 129 a	5616 ± 136 a	5425 ± 131 a	5431 ± 131 a	0.538	0.552	0.379
**CD99**	CD99 molecule (Xg blood group)	9298 ± 260 ac	9410 ± 263 a	9539 ± 267 a	8610 ± 241 bc	8650 ± 242 bc	8315 ± 233 b	0.890	0.000	0.490
**CDC42**	cell division cycle 42	19726 ± 364 a	20004 ± 369 a	19961 ± 368 a	21578 ± 398 b	20567 ± 379 ab	20718 ± 382 ab	0.599	0.002	0.197
**CDK4**	cyclin dependent kinase 4	2486 ± 141 a	2699 ± 153 ab	3084 ± 174 b	1677 ± 95 c	2374 ± 134 a	2626 ± 149 a	0.000	0.000	0.045
**CGN**	cingulin	1531 ± 126 bc	1423 ± 117 ab	1329 ± 110 ab	1469 ± 121 b	1870 ± 154 c	1149 ± 95 a	0.006	0.665	0.039
**CLDN1**	claudin 1	10369 ± 408 b	9586 ± 377 ab	9071 ± 357 ad	8306 ± 327 cd	8050 ± 317 c	6603 ± 260 e	0.000	0.000	0.183
**CLDN10**	claudin 10	39 ± 4 a	40 ± 4 a	35 ± 3 a	40 ± 4 a	39 ± 4 a	33 ± 3 a	0.160	0.801	0.916

**CLDN11**	claudin 11	38 ± 3 b	42 ± 3 ab	41 ± 3 ab	45 ± 3 ab	47 ± 3 a	47 ± 3 a	0.537	0.010	0.950
**CLDN12**	claudin 12	773 ± 21 ab	796 ± 22 a	765 ± 21 ab	746 ± 20 ab	769 ± 21 ab	731 ± 20 b	0.236	0.081	0.971
**CLDN14**	claudin 14	30 ± 3 a	26 ± 2 ab	23 ± 2 b	32 ± 3 a	31 ± 3 a	27 ± 2 ab	0.037	0.098	0.832
**CLDN15**	claudin 15	204 ± 9 a	202 ± 9 a	198 ± 9 a	196 ± 8 a	237 ± 10 b	186 ± 8 a	0.012	0.619	0.023
**CLDN16**	claudin 16	347 ± 16 a	315 ± 15 ab	324 ± 15 ab	291 ± 13 bc	270 ± 12 c	233 ± 11 d	0.011	0.000	0.126
**CLDN17**	claudin 17	34 ± 4 a	31 ± 3 a	28 ± 3 a	36 ± 4 a	29 ± 3 a	31 ± 3 a	0.160	0.720	0.721
**CLDN18**	claudin 18	23 ± 2 ac	20 ± 2 a	18 ± 2 a	29 ± 3 bc	31 ± 3 b	18 ± 2 a	0.006	0.012	0.149
**CLDN19**	claudin 19	70 ± 4 ab	73 ± 4 a	68 ± 3 ab	62 ± 3 b	70 ± 4 ab	63 ± 3 ab	0.204	0.082	0.741
**CLDN2**	claudin 2	561 ± 25 b	606 ± 27 ab	766 ± 34 d	420 ± 19 e	487 ± 22 c	643 ± 28 a	0.000	0.000	0.426
**CLDN20**	claudin 20	24 ± 2 bd	28 ± 2 ab	23 ± 2 d	34 ± 2 c	33 ± 2 ac	25 ± 2 bd	0.004	0.001	0.140
**CLDN3**	claudin 3	1959 ± 66 a	1836 ± 62 a	1622 ± 55 c	2918 ± 99 d	2425 ± 82 b	1933 ± 65 a	0.000	0.000	0.009
**CLDN4**	claudin 4	25290 ± 1152 b	21882 ± 997 a	17639 ± 804 c	32873 ± 1497 d	28596 ± 1303 b	21022 ± 958 a	0.000	0.000	0.519
**CLDN5**	claudin 5	23 ± 2 c	17 ± 1 ab	14 ± 1 b	17 ± 1 ab	21 ± 2 ac	16 ± 1 b	0.012	0.735	0.017
**CLDN6**	claudin 6	130 ± 19 a	111 ± 17 a	122 ± 18 a	115 ± 17 a	95 ± 14 a	87 ± 13 a	0.401	0.104	0.719
**CLDN7**	claudin 7	7518 ± 175 a	7498 ± 174 a	7994 ± 186 ac	7548 ± 176 a	8578 ± 200 b	8126 ± 189 bc	0.011	0.010	0.015
**CLDN8**	claudin 8	15 ± 2 a	14 ± 2 a	14 ± 2 a	14 ± 2 a	17 ± 2 a	15 ± 2 a	0.859	0.618	0.630
**CLDN9**	claudin 9	38 ± 3 ab	39 ± 3 ab	39 ± 3 ab	43 ± 3 a	40 ± 3 a	33 ± 2 b	0.199	0.863	0.077
**CRB1**	CNKSR family member 3	37 ± 3 bc	34 ± 3 ab	28 ± 3 a	41 ± 4 bc	48 ± 4 c	40 ± 4 bc	0.092	0.001	0.272
**CRB3**	crumbs cell polarity complex component 1	3139 ± 92 a	3175 ± 93 a	3043 ± 89 a	3804 ± 111 c	3462 ± 101 b	3138 ± 92 a	0.002	0.000	0.028
**CSNK2A1**	crumbs cell polarity complex component 3	83 ± 6 a	92 ± 7 a	91 ± 7 a	132 ± 10 b	126 ± 9 b	118 ± 9 b	0.853	0.000	0.351

**CSNK2A2**	casein kinase 2 alpha 2	125 ± 13 a	120 ± 13 ab	103 ± 11 ab	90 ± 9 b	135 ± 14 a	91 ± 10 b	0.038	0.191	0.097
**CSNK2B**	casein kinase 2 alpha 2	9498 ± 173 bc	9303 ± 170 ab	8885 ± 162 a	9846 ± 180 c	9859 ± 180 c	9080 ± 166 ab	0.000	0.013	0.600
**CTNNA1**	casein kinase 2 beta	15336 ± 479 a	14879 ± 465 a	14295 ± 447 a	16793 ± 525 b	17121 ± 535 b	14736 ± 460 a	0.004	0.002	0.214
**CTNNA2**	catenin alpha 1	25 ± 4 ac	19 ± 3 ab	12 ± 2 b	32 ± 6 c	21 ± 4 ac	22 ± 4 ac	0.007	0.021	0.358
**CTNNA3**	catenin alpha 2	23 ± 3 b	17 ± 3 ab	14 ± 2 a	22 ± 3 b	20 ± 3 ab	18 ± 3 ab	0.051	0.316	0.664
**CTNNB1**	catenin alpha 3	5374 ± 159 a	5176 ± 153 a	4951 ± 146 a	6124 ± 181 b	6063 ± 179 b	5198 ± 153 a	0.001	0.000	0.162
**CTTN**	catenin beta 1	9905 ± 440 ab	9470 ± 421 ab	10379 ± 461 a	9112 ± 405 b	10711 ± 476 a	10394 ± 462 a	0.130	0.704	0.072
**EPB41**	cortactin	436 ± 15 ab	406 ± 14 a	434 ± 15 ab	443 ± 15 ab	461 ± 16 b	416 ± 14 a	0.619	0.240	0.049
**ESAM**	erythrocyte membrane protein band 4.1	335 ± 15 a	356 ± 16 a	338 ± 15 a	470 ± 21 c	446 ± 20 bc	410 ± 18 b	0.231	0.000	0.224
**EZR**	endothelial cell adhesion molecule	13905 ± 455 a	12890 ± 421 a	11464 ± 375 c	20249 ± 662 d	16831 ± 550 b	12740 ± 416 a	0.000	0.000	0.001
**F11R**	ezrin	7640 ± 228 b	7315 ± 218 ab	6875 ± 205 a	9023 ± 269 c	9026 ± 269 c	7506 ± 224 b	0.000	0.000	0.124
**GNAI1**	F11 receptor	66 ± 4 ab	67 ± 4 ab	57 ± 3 a	68 ± 4 b	60 ± 4 ab	42 ± 2 c	0.000	0.007	0.021
**HCLS1**	G protein subunit alpha i1	27 ± 3 a	33 ± 4 a	28 ± 3 a	35 ± 4 a	26 ± 3 a	27 ± 3 a	0.592	0.935	0.143
**ICAM1**	hematopoietic cell-specific Lyn substrate 1	2419 ± 90 a	2375 ± 88 a	1742 ± 65 c	3001 ± 111 b	3021 ± 112 b	1891 ± 70 c	0.000	0.000	0.081
**ICAM2**	intercellular adhesion molecule 1	102 ± 6 a	102 ± 6 a	93 ± 5 a	142 ± 8 b	143 ± 8 b	132 ± 7 b	0.214	0.000	0.983
**IGSF5**	intercellular adhesion molecule 2	42 ± 3 d	52 ± 4 abc	44 ± 3 bd	62 ± 4 ac	62 ± 4 a	51 ± 4 bcd	0.043	0.000	0.205
**ILK**	immunoglobulin superfamily member 5	3556 ± 58 a	3590 ± 58 a	3396 ± 55 c	3880 ± 63 b	3812 ± 62 b	3534 ± 57 ac	0.000	0.000	0.337
**JAM2**	integrin linked kinase	12 ± 1 a	11 ± 1 a	10 ± 1 a	12 ± 1 a	11 ± 1 a	11 ± 1 a	0.254	0.543	0.934
**JAM3**	junctional adhesion molecule 2	32 ± 3 a	36 ± 4 a	28 ± 3 a	37 ± 4 a	37 ± 4 a	30 ± 3 a	0.079	0.314	0.851
**LLGL1**	junctional adhesion molecule 3	623 ± 15 a	626 ± 15 a	636 ± 15 a	552 ± 13 b	641 ± 15 a	650 ± 15 a	0.001	0.174	0.005

**LLGL2**	LLGL scribble cell polarity complex component 1	1606 ± 98 a	1595 ± 98 a	1494 ± 92 a	1507 ± 92 a	1935 ± 119 b	1466 ± 90 a	0.023	0.457	0.090
**MAGI1**	LLGL scribble cell polarity complex component 2	423 ± 30 ab	405 ± 29 ab	394 ± 28 ab	413 ± 30 ab	443 ± 32 a	346 ± 25 b	0.113	0.707	0.316
**MAGI2**	ligand of numb-protein X 1	25 ± 2 ab	24 ± 2 ab	19 ± 2 b	31 ± 3 a	27 ± 3 a	26 ± 2 a	0.077	0.006	0.587
**MARK2**	membrane associated guanylate kinase, WW and PDZ domain containing 2	3146 ± 98 bc	3063 ± 95 ab	2817 ± 88 a	3562 ± 111 d	3349 ± 104 cd	3011 ± 94 ab	0.000	0.001	0.638
**MARVELD2**	microtubule affinity regulating kinase 2	2297 ± 106 b	2185 ± 101 ab	1805 ± 83 c	1933 ± 89 ac	1928 ± 89 ac	1527 ± 70 d	0.000	0.000	0.845
**MPDZ**	MARVEL domain containing 2	27 ± 3 a	33 ± 3 a	29 ± 3 a	33 ± 3 a	32 ± 3 a	32 ± 3 a	0.613	0.235	0.472
**MPP5**	occludin	2881 ± 86 ab	2815 ± 84 ab	2584 ± 78 c	2936 ± 88 b	2673 ± 80 ac	2269 ± 68 d	0.000	0.031	0.056
**MPP6**	protein associated with LIN7 1, MAGUK p55 family member	890 ± 25 b	1009 ± 28 a	1160 ± 32 c	882 ± 24 b	952 ± 26 ab	1220 ± 34 c	0.000	0.807	0.152
**OCLN**	protein associated with LIN7 2, MAGUK p55 family member	2382 ± 76 d	2164 ± 69 a	1814 ± 58 b	2215 ± 71 ad	2111 ± 67 a	1538 ± 49 c	0.000	0.002	0.094
**PARD3**	par-3 family cell polarity regulator	3638 ± 104 a	3599 ± 103 a	3178 ± 91 c	4345 ± 125 b	4141 ± 119 b	3404 ± 98 ac	0.000	0.000	0.162
**PARD6A**	par-6 family cell polarity regulator alpha	98 ± 6 a	104 ± 6 a	103 ± 6 a	99 ± 6 a	102 ± 6 a	103 ± 6 a	0.663	1.000	0.987
**PARD6B**	par-6 family cell polarity regulator beta	1017 ± 57 ac	905 ± 51 ab	826 ± 46 b	1136 ± 64 c	1020 ± 57 ac	659 ± 37 d	0.000	0.969	0.005
**PECAM1**	platelet and endothelial cell adhesion molecule 1	58 ± 4 a	53 ± 4 a	56 ± 4 a	53 ± 4 a	51 ± 4 ab	42 ± 3 b	0.250	0.024	0.268
**PRKCI**	protein kinase C iota	2036 ± 47 bc	1957 ± 45 ab	1798 ± 42 d	2331 ± 54 e	2091 ± 48 c	1833 ± 42 ad	0.000	0.000	0.050
**PRKCZ**	protein kinase C zeta	1365 ± 46 ab	1351 ± 45 ab	1325 ± 44 ac	1459 ± 49 b	1449 ± 49 ab	1228 ± 41 c	0.007	0.459	0.055
**PTEN**	phosphatase and tensin homolog	3843 ± 132 b	3785 ± 130 ab	3304 ± 114 c	4007 ± 138 b	3479 ± 120 ac	2993 ± 103 d	0.000	0.095	0.089
**RAC1**	radixin	7428 ± 187 a	7260 ± 183 a	6484 ± 163 c	8586 ± 216 b	8899 ± 224 b	7545 ± 190 a	0.000	0.000	0.436
**RDX**	ras homolog family member A	12288 ± 523 a	12308 ± 524 a	12456 ± 530 a	11355 ± 484 ab	10345 ± 441 b	10283 ± 438 b	0.481	0.000	0.363

**RHOA**	ribonuclease A family member 1, pancreatic	3947 ± 106 a	3939 ± 105 a	3980 ± 106 a	3823 ± 102 a	3888 ± 104 a	3722 ± 100 a	0.817	0.089	0.584
**ROCK1**	Rho associated coiled-coil containing protein kinase 1	1641 ± 69 ac	1572 ± 66 ab	1477 ± 62 ab	1810 ± 76 c	1625 ± 68 ac	1398 ± 59 b	0.001	0.449	0.191
**SMURF1**	SMAD specific E3 ubiquitin protein ligase 1	570 ± 22 a	547 ± 21 a	491 ± 19 c	788 ± 30 d	702 ± 27 b	559 ± 21 a	0.000	0.000	0.047
**SPTA1**	spectrin alpha, erythrocytic 1	10 ± 2 a	12 ± 2 a	10 ± 2 a	12 ± 2 a	14 ± 2 a	11 ± 2 a	0.472	0.239	0.891
**SPTAN1**	spectrin alpha, non-erythrocytic 1	4063 ± 219 ab	3720 ± 201 a	3843 ± 208 ab	4305 ± 233 ab	4402 ± 238 b	3794 ± 205 ab	0.234	0.108	0.240
**SPTB**	spectrin beta, erythrocytic	42 ± 2 b	36 ± 2 a	29 ± 2 c	38 ± 2 ab	41 ± 2 ab	37 ± 2 ab	0.003	0.043	0.006
**SYMPK**	symplekin scaffold protein	2933 ± 157 a	2838 ± 152 a	2742 ± 147 a	2738 ± 147 a	2880 ± 155 a	2598 ± 139 a	0.374	0.401	0.700
**TIAM1**	TIAM Rac1 associated GEF 1	68 ± 5 b	59 ± 5 ab	52 ± 4 ac	46 ± 4 c	48 ± 4 ac	42 ± 3 c	0.071	0.000	0.345
**TJAP1**	tight junction associated protein 1	397 ± 15 ac	430 ± 16 a	377 ± 14 c	503 ± 19 b	503 ± 19 b	401 ± 15 ac	0.000	0.000	0.071
**TJP1**	tight junction protein 1	2850 ± 134 a	2680 ± 126 a	2549 ± 120 a	3972 ± 187 c	3406 ± 161 b	2599 ± 123 a	0.000	0.000	0.006
**TJP2**	tight junction protein 2	3359 ± 224 a	3174 ± 212 a	2913 ± 194 a	4337 ± 289 b	4393 ± 293 b	2953 ± 197 a	0.001	0.001	0.058
**TJP3**	tight junction protein 3	2761 ± 98 a	2790 ± 99 a	2767 ± 99 a	3552 ± 127 b	3602 ± 128 b	2836 ± 101 a	0.002	0.000	0.003
**VAPA**	VAMP associated protein A	10631 ± 177 ab	10277 ± 171 a	9453 ± 158 c	12029 ± 201 d	10867 ± 181 b	9285 ± 155 c	0.000	0.000	0.001
**WAS**	WASP actin nucleation promoting factor	39 ± 3 b	34 ± 3 ab	32 ± 3 ab	34 ± 3 ab	30 ± 2 ac	25 ± 2 c	0.012	0.008	0.540
**WASL**	WASP like actin nucleation promoting factor	5759 ± 222 ac	5490 ± 212 a	4769 ± 184 b	6147 ± 237 c	5343 ± 206 a	4288 ± 165 b	0.000	0.463	0.093
**WHAMM**	WASP homolog associated with actin, golgi membranes and microtubules	839 ± 33 b	808 ± 32 ab	735 ± 29 a	1157 ± 45 d	989 ± 39 c	789 ± 31 ab	0.000	0.000	0.011
**YBX3**	Y-box binding protein 3	2826 ± 82 a	2919 ± 85 a	2798 ± 81 a	3613 ± 105 b	3521 ± 102 b	3238 ± 94 c	0.060	0.000	0.225

[1] Mean (± SEM) normalised gene expression for each treatment-timepoint combination is presented (n=6 per treatment per timepoint). Treatments which do not share the same letter (a, b, c, d) are significantly different ($P<0.05$).
[2] Model used for the permutation ANOVAs was gene ~ experiment + treatment + timepoint + treatment×timepoint
